# Supplementary figures and images for: Single-molecule dynamics and genome-wide transcriptomics reveal that NF-kB (p65)-DNA binding times can be decoupled from transcriptional activation
Source: PLoS Genet. 2019 Jan 17;15(1):e1007891. doi: 10.1371/journal.pgen.1007891 (PMC6353211; doi:10.1371/journal.pgen.1007891)

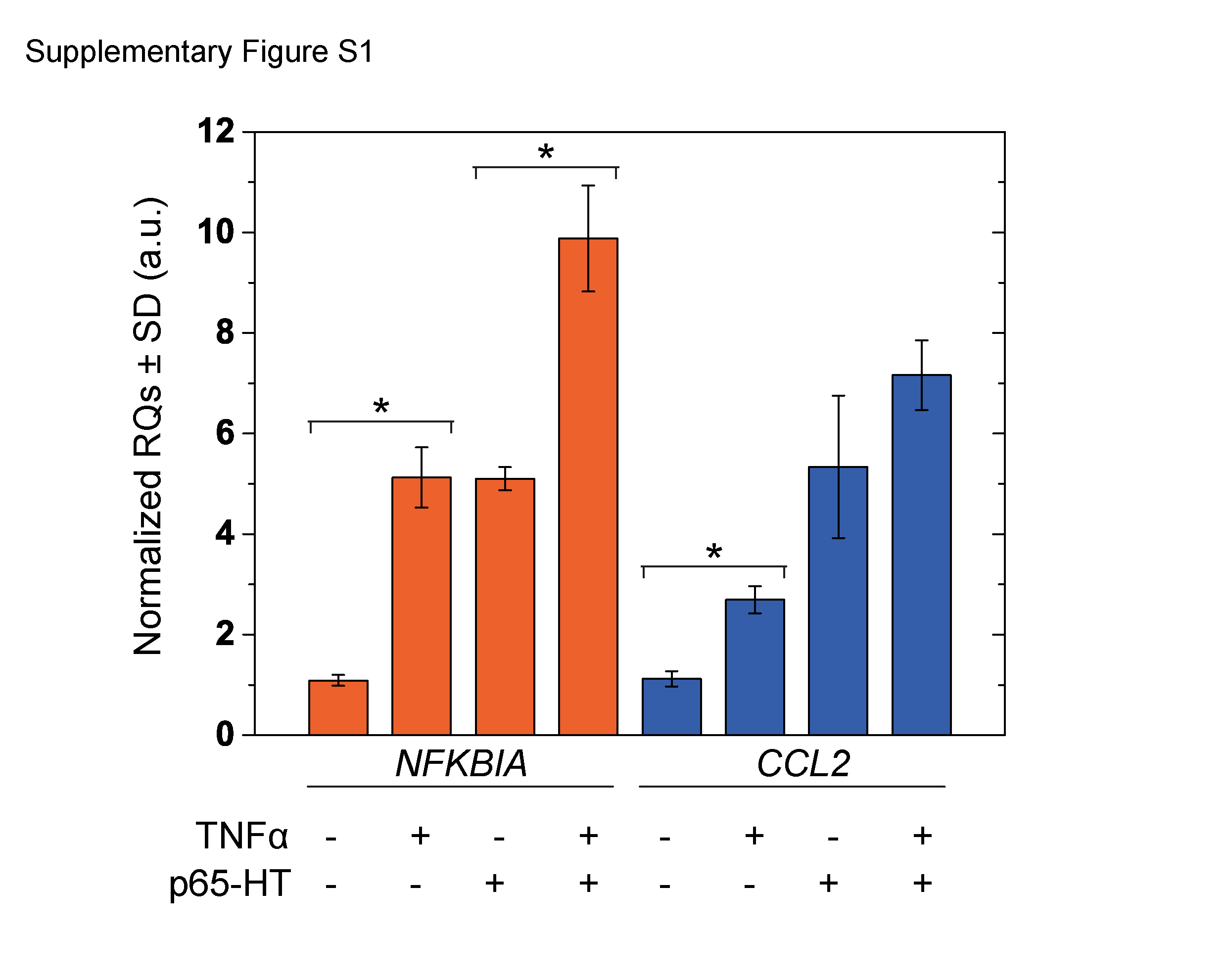

Supplement: S1 Fig — Averages of normalized relative quantities (RQs) of biological triplicates ± standard deviation (SD) are shown. * p < 0.05. (TIF) [file pgen.1007891.s001.tif]

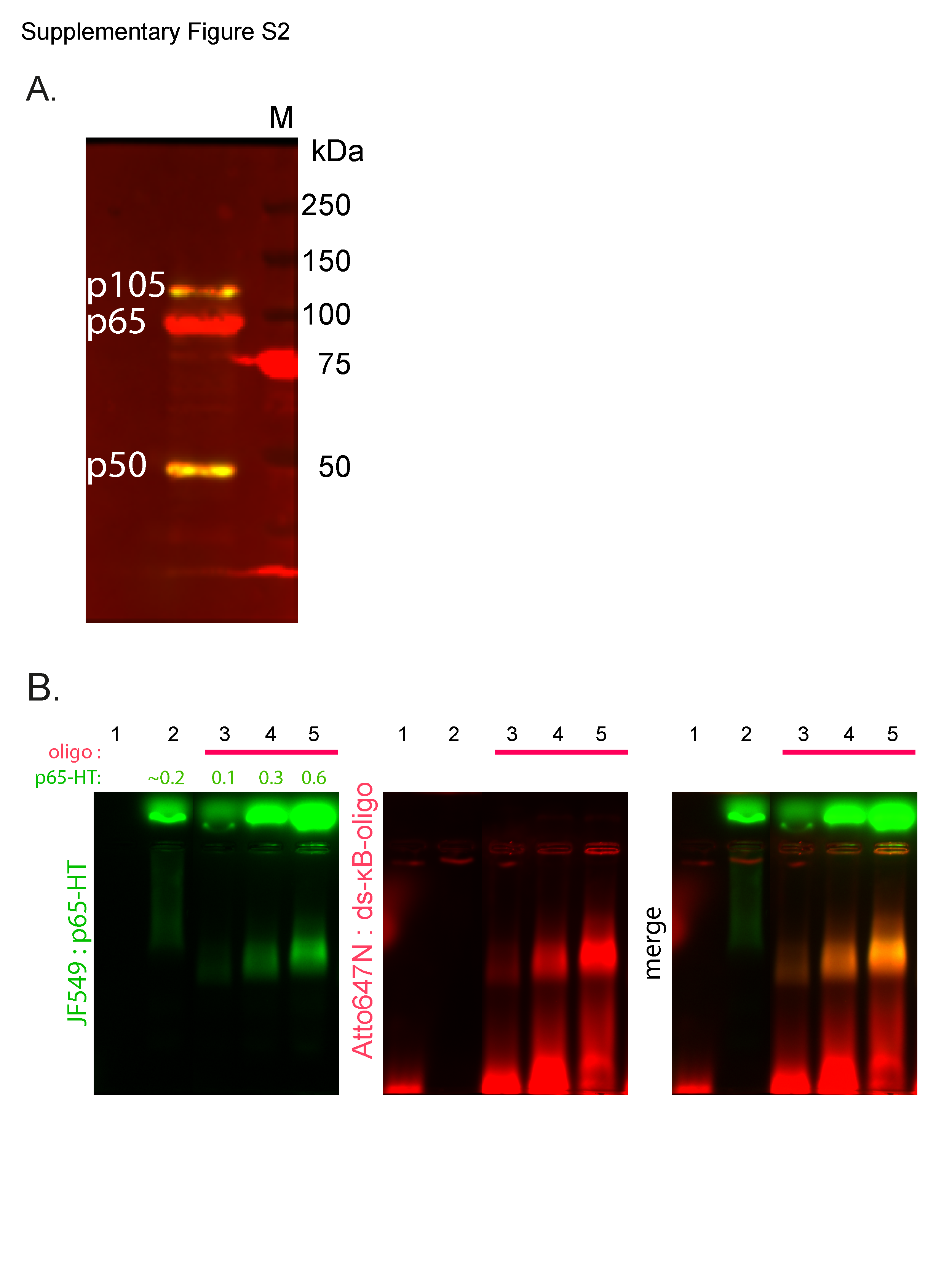

Supplement: S2 Fig — (A) Western blot of p65-Halo purified fraction. Merged signals out of anti-p65 and anti-p50 are shown. p105 corresponds to the precursor protein that is subsequently cleaved into p50 and p65. (B) Electrophoretic mobility shift assay (EMSA) of wild-type p65-Halo-tagged construct (p65-HT) and its consensus oligo (ds-κB-oligo). Increasing quantities (in μg) of JF549-labeled p65-HT (green channel) and a constant amount of ds-κB-oligo (red channel) are incubated together and electrophoretically separated under native conditions. The merge of both channels shows overlapping p65-HT fluorescence with consensus oligo signal. (TIF) [file pgen.1007891.s002.tif]

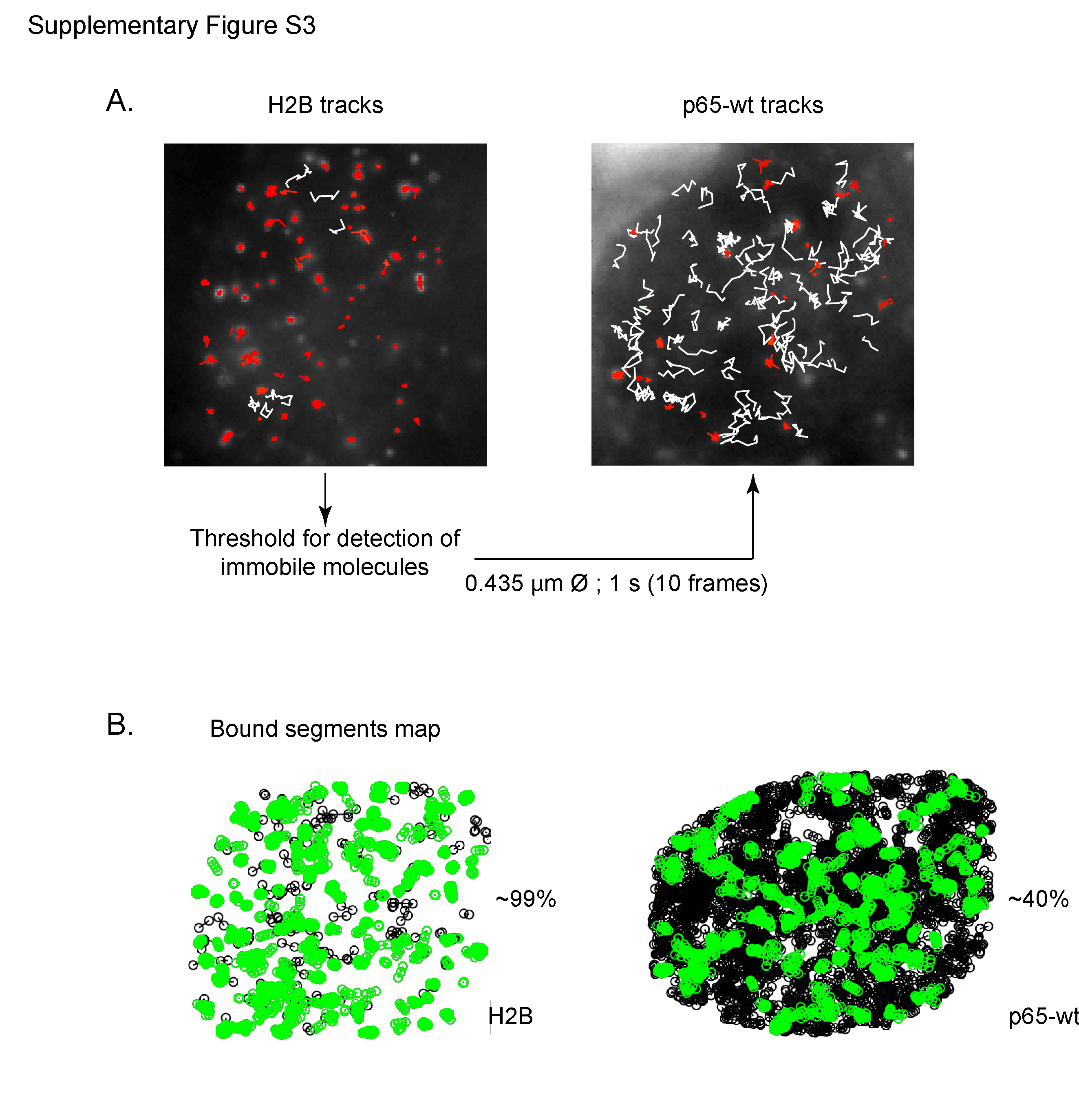

Supplement: S3 Fig — (A) Binding events of individual p65 molecules are detected based on both spatial (435 nm) and temporal (1 s) thresholds experimentally established from imaging of immobile H2B molecules. (B) Analysis of bound segments of H2B (left) and p65-WT (right), using the spatiotemporal criteria explained in panel A, assigns ~99% of H2B molecules and ~40% of p65-WT to the ‘bound’ state. (TIF) [file pgen.1007891.s003.tif]

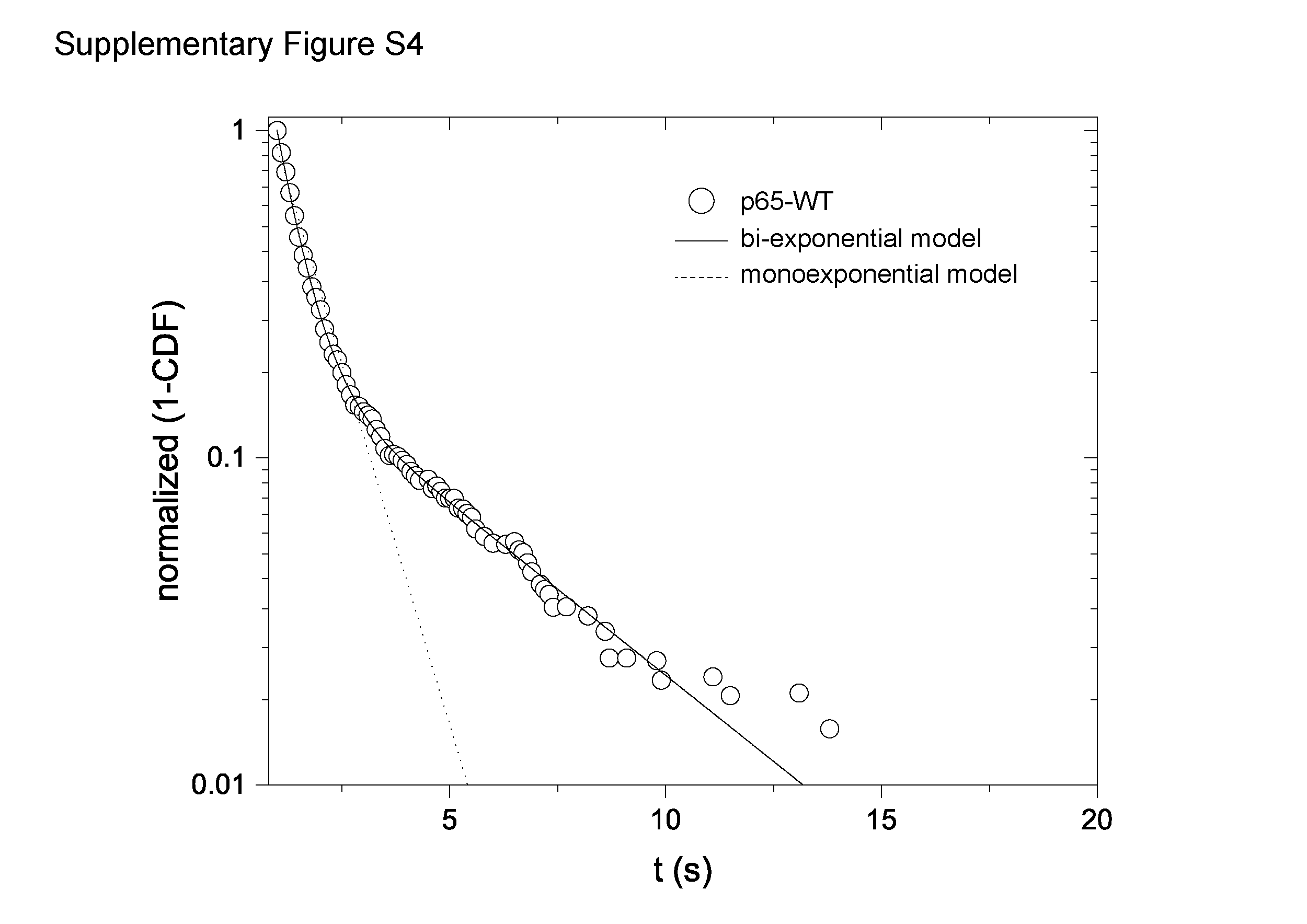

Supplement: S4 Fig — (TIF) [file pgen.1007891.s004.tif]

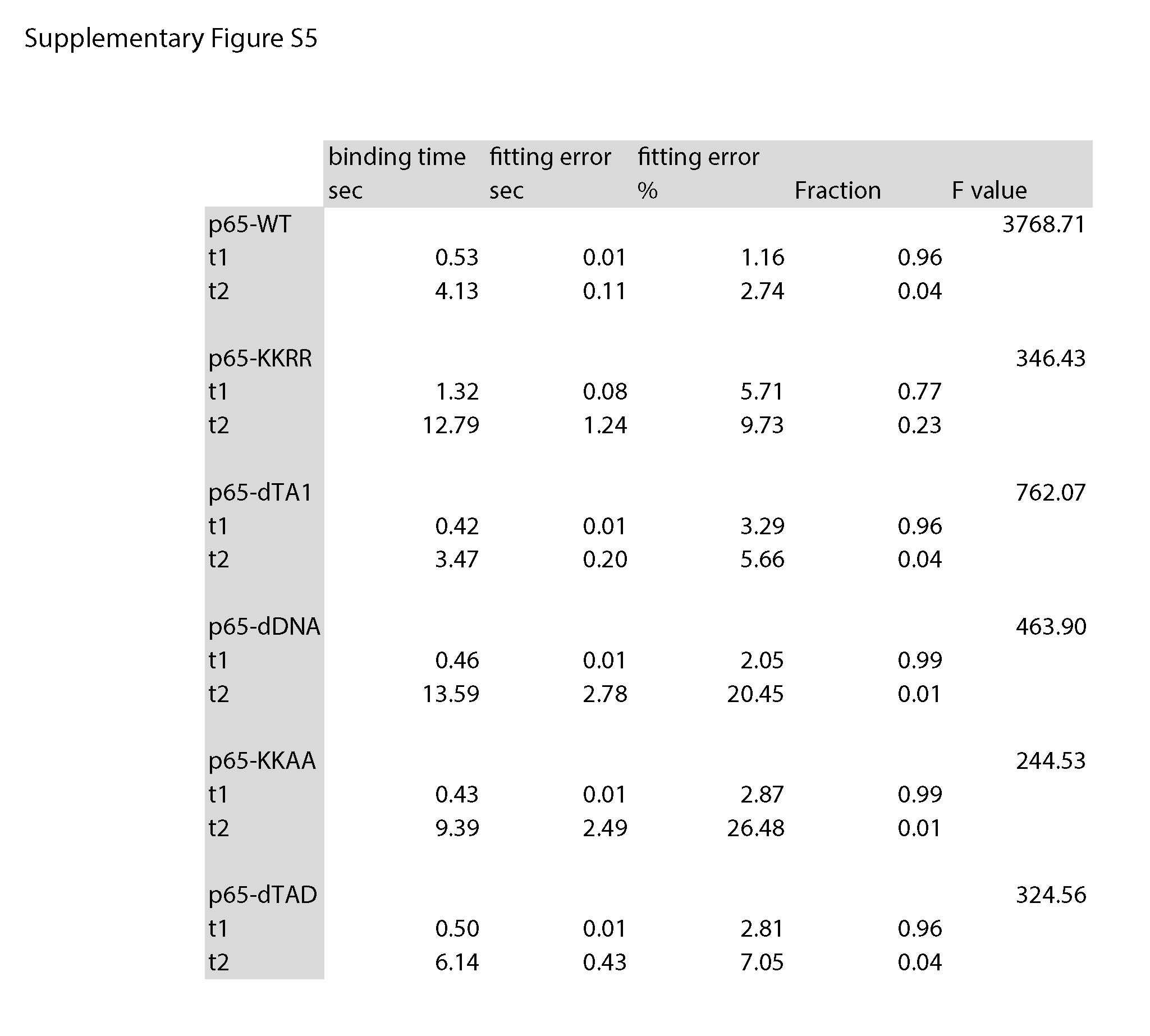

Supplement: S5 Fig — We performed mono- and bi-exponential fitting as well as an F-Test (function ‘compare models’ in Origin Pro 2018) to compare the quality of the two fitting models in describing the normalized survival probability distributions. The fitting parameters for tbfast (t1) and tbslow (t2) as well as their associated error are shown together with the respective fraction and the F value for each p65 mutant. (TIF) [file pgen.1007891.s005.tif]

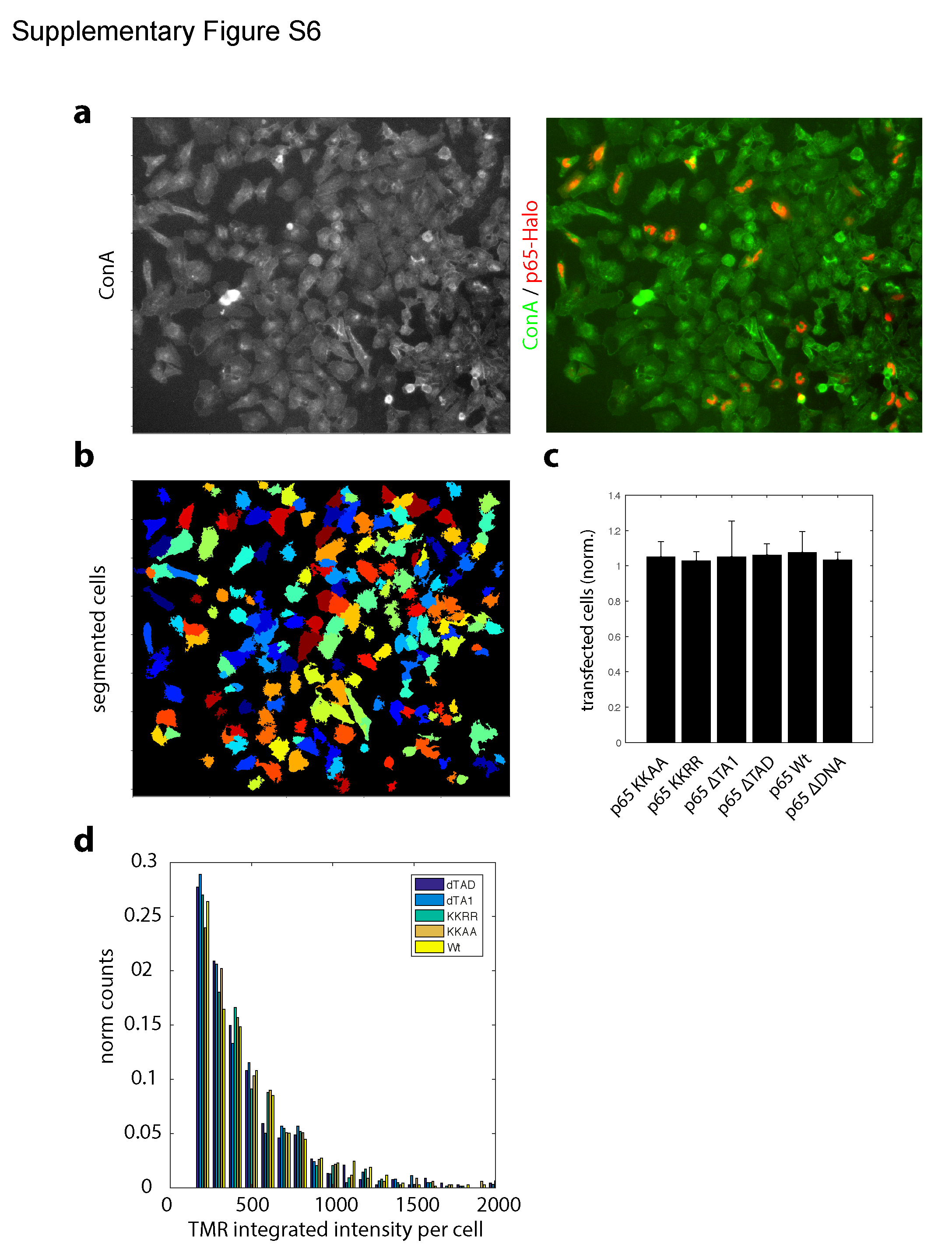

Supplement: S6 Fig — Hela cells where transfected with p65 mutants under identical conditions, labelled with TMR-Halo and analyzed using fluorescence microscopy (A). Concavalin A conjugated to Alexa647 was used as a cell marker to allow automatic segmentation (B). From the fluorescence micrographs of ConA (A), individual cells where segmented using Cellprofiler (B). The TMR signal was quantified per cell and cells with a markedly higher TMR staining where counted as transfected and normalized against the number of cells per field of view. The transfection levels were then again normalized per experiment. Shown are mean and SD of three independent experiments (C). (D) shows a histogram of the integrated TMR intensity per cell. (TIF) [file pgen.1007891.s006.tif]

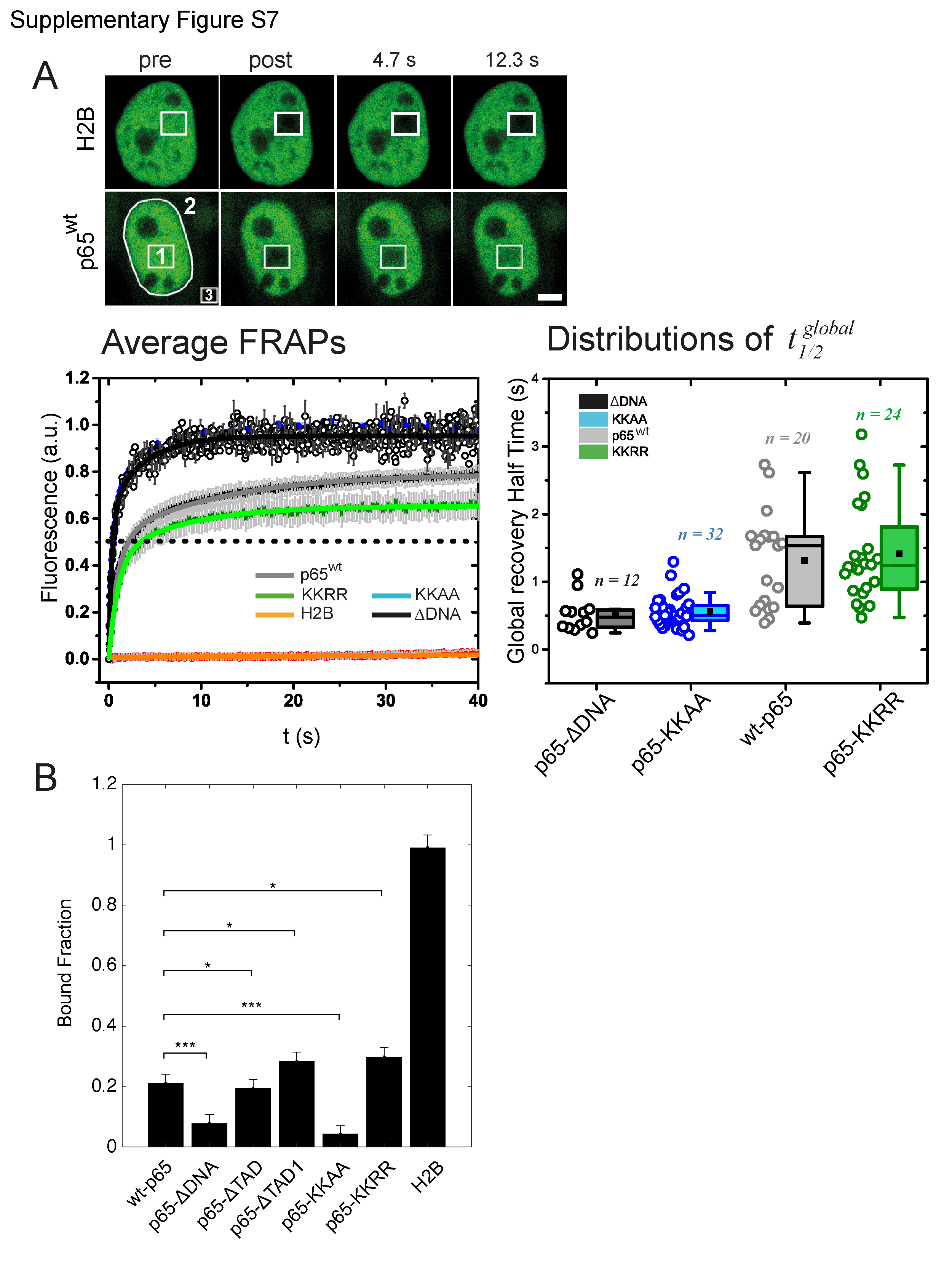

Supplement: S7 Fig — (A) Fluorescence recovery after photobleaching (FRAP) of p65-WT and its DNA-binding affinity mutants. Pre- and post-bleaching snapshots of a representative nucleus overexpressing the H2B-Halo construct (top). The actual size of the bleached region is highlighted with a white rectangle. Note that H2B-Halo fluorescence does not recover, confirming that H2B-Halo is immobile in living Hela cells. Different regions of interest (ROIs) used to calculate the FRAP recovery curves are indicated with numbers (1, 2 and 3; low). 1: bleaching ROI; 2: reference ROI encompassing the whole nuclear area used to normalize against the actual expression levels and photobleaching; 3: background. Representative time-points of p65-WT fluorescence recovery are shown. Scale bar: 5 μm. Averaged normalized FRAP curves of p65-WT, DNA-binding affinity mutants and ΔDNA (control) collected from Hela cells stimulated as described in Methods (left). Curves obtained from double-exponential model fitting of experimental data-points (see Methods) are superimposed to estimate t1/2global. Distributions of t1/2global are represented as box-plots (right). The average (black square) and the median values (horizontal line) of each distribution are displayed for each box-plot together with the number (n) of measured cell nuclei. Whiskers span over the 25%-75% percentile range. (B) Analysis of p65 bound fraction (BF). Histogram of the BF values of p65-WT and its mutants compares each construct to H2B bound-fraction. * p < 0.05, ***p<0.001. (TIF) [file pgen.1007891.s007.tif]

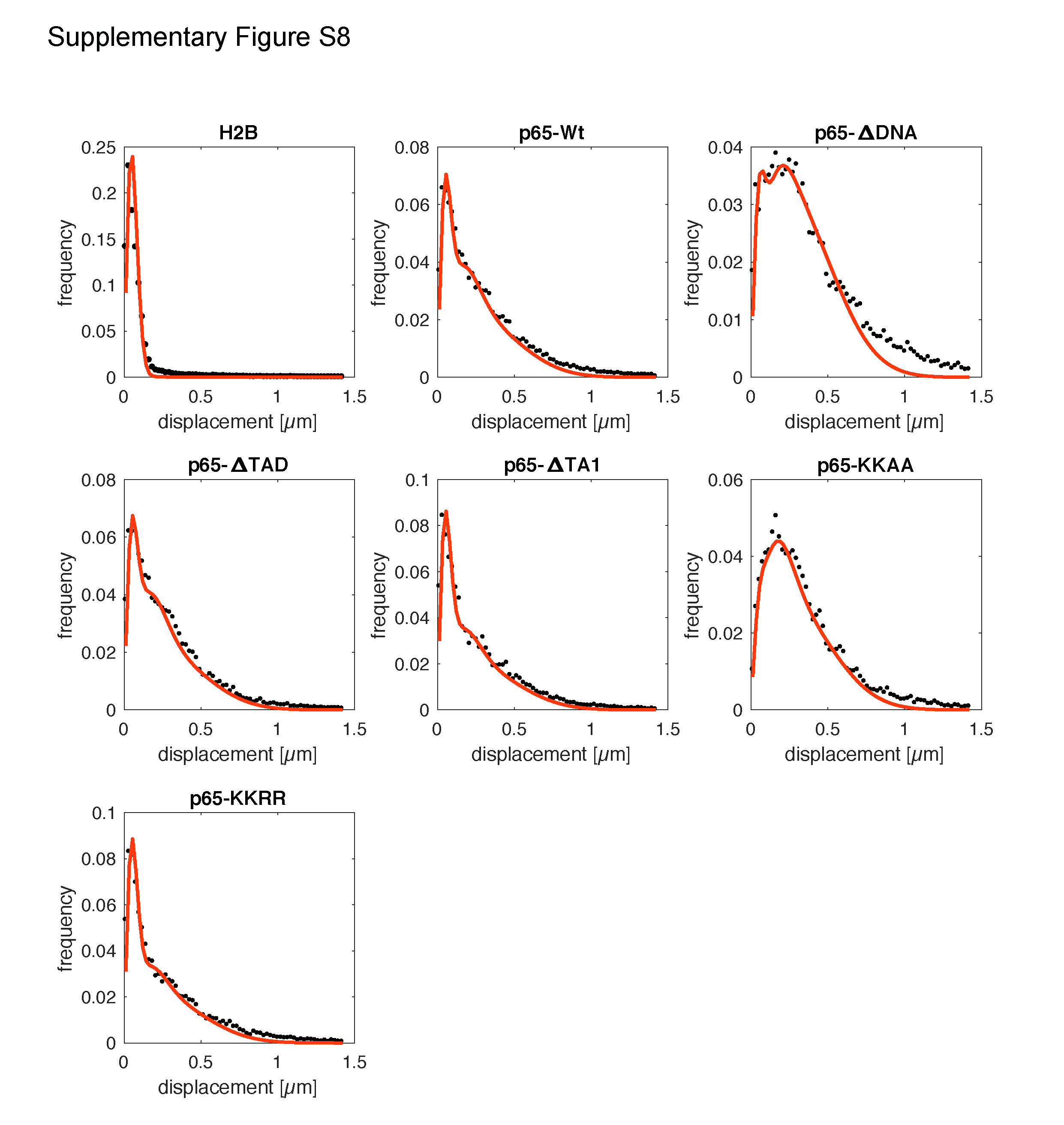

Supplement: S8 Fig — The diffusion constant calculated for the DNA-bound H2B histone subunit matched the slowest diffusing component of the p65 construct whose amplitude corresponds to the computed BF. (TIF) [file pgen.1007891.s008.tif]

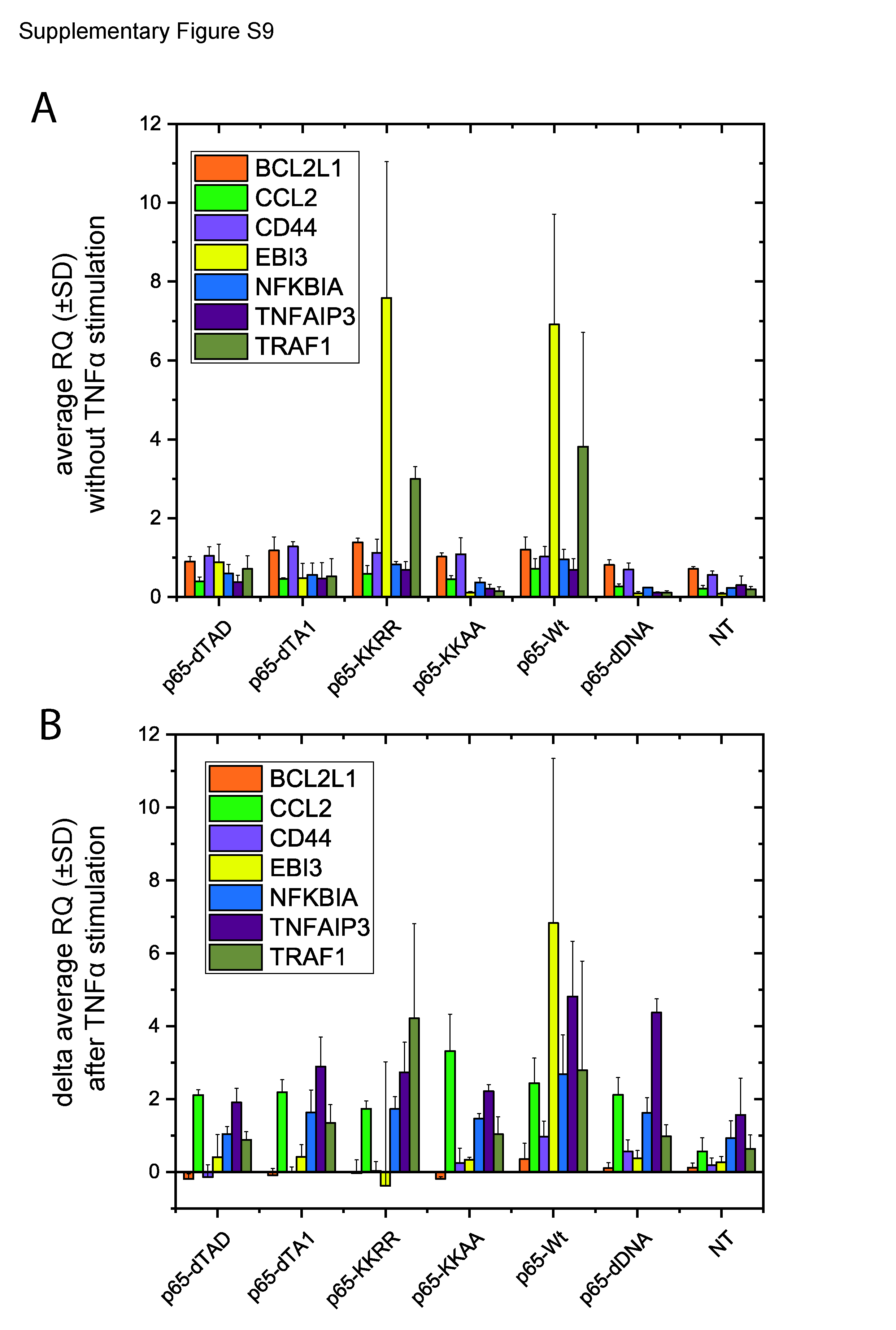

Supplement: S9 Fig — Extended qPCR analysis of Hela cells transfected with the respective p65 variant testing for spontaneous activation (A) and sensitivity to TNF-α (B). In addition to NFKBIA and CCL2 (S1 Fig), we tested a small subset of 5 genes shown to be regulated by NFkB and recognized in our RNA-seq analysis. Averages of normalized relative quantities (RQs) of biological triplicates ± standard deviation (SD) are shown. (TIF) [file pgen.1007891.s009.tif]

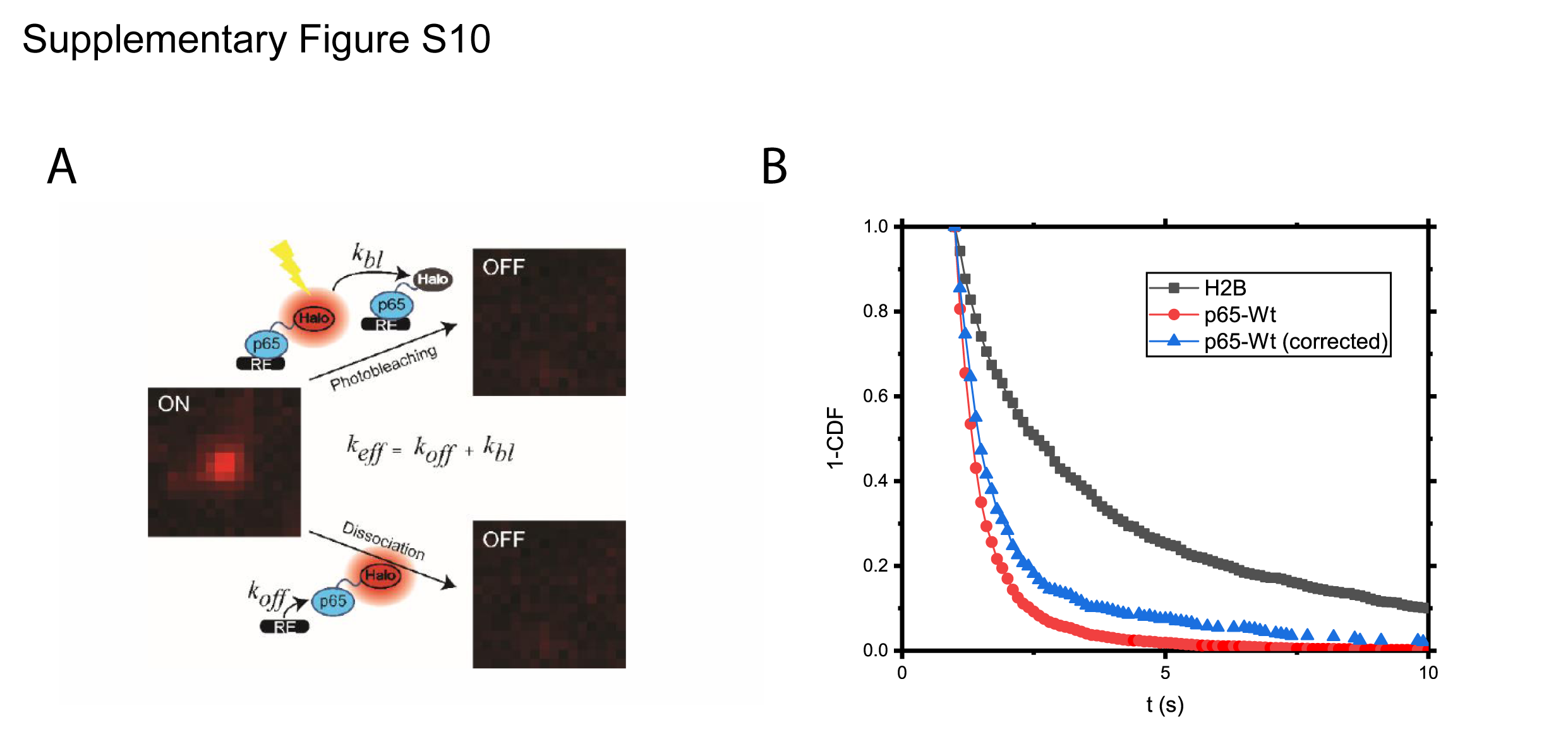

Supplement: S10 Fig — In order to assess koff, the effective dissociation rate needs to be corrected by the photobleaching rate (A). To this end, the survival probability of H2B was measured and plotted using the same procedure as described for p65. Note that the 1-CDF histogram of H2B decays at a much slower rate compared to p65 (B). This photobleaching rate can then be used to correct the 1-CDF histogram of p65 using Eq 1. (TIF) [file pgen.1007891.s010.tif]

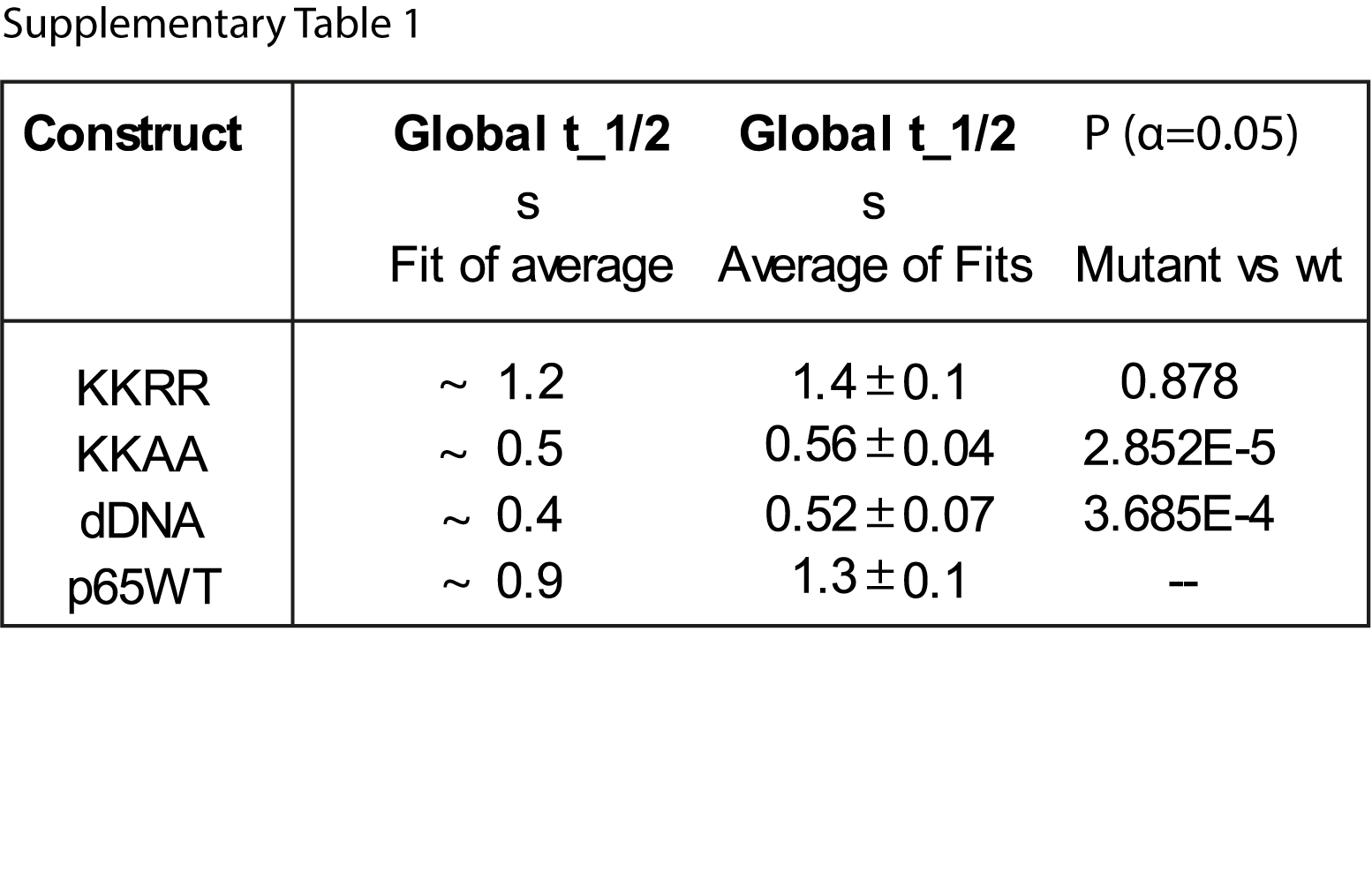

Supplement: S1 Table — (TIF) [file pgen.1007891.s011.tif]
